# ^99m^Tc-^68^Ga-ICG-Labelled Macroaggregates and Nanocolloids of Human Serum Albumin: Synthesis Procedures of a Trimodal Imaging Agent Using Commercial Kits

**DOI:** 10.1155/2020/3629705

**Published:** 2020-01-22

**Authors:** Marco Giovanni Persico, Manuela Marenco, Gianluca De Matteis, Giulia Manfrinato, Giorgio Cavenaghi, Adele Sgarella, Carlo Aprile, Lorenzo Lodola

**Affiliations:** ^1^Fondazione IRCCS Policlinico San Matteo, Nuclear Medicine Unit, 27100 Pavia, Italy; ^2^University School for Advanced Studies IUSS, 27100 Pavia, Italy; ^3^Fondazione IRCCS Policlinico San Matteo, Breast Cancer Unit, 27100 Pavia, Italy; ^4^National Center for Oncological Hadrontherapy (CNAO), 27100 Pavia, Italy

## Abstract

Recent developments in sentinel lymph node (SLN) and radio occult lesion localization (ROLL) highlight the need for a multimodal contrast agent, providing better presurgical PET imaging and improved intraoperative mapping thanks to fluorescence detection. For this reason, we have studied a trimodal SLN/ROLL targeting agent (^99m^Tc-^68^Ga-ICG) with commercially available kits of macroaggregated or nanocolloidal albumin (MA/NC-HSA). ^68^Ga PET imaging does provide better spatial resolution and makes it possible to predict signal intensity during surgery. The presence of ^99m^Tc assesses the efficacy of these compounds *in vitro* and also during the surgery procedure. The aim of this study was to optimise the labelling and tagging of these two radiopharmaceuticals and assess their yields and stability. Kits of MA/NC-HSA particles (Pulmocis® and NanoAlbumon®) were used for sequential radiolabelling with ^99m^Tc and ^68^Ga. Fluorescent tagging was performed using indocyanine green, a tricarbocyanine dye. The ITLC radiochemical purity of the trilabelled MA/NC-HSA was >95%. Fluorescent purity was measured by scanning the strips with a PhotoDynamicEye probe. Finally, *in vitro* stability tests, performed with DTPA and human serum solutions, assessed the efficacy of fluorescent tagging and radiolabelling.

## 1. Introduction

Recent developments in sentinel lymph node (SLN), radioguided occult lesion localization (ROLL) in the mapping of breast and other cancers, as well as evaluating regional perfusion in healthy and cancerous tissue, points to the need for a multimodal contrast agent that can provide both better cross-sectional images for presurgical planning via positron emission tomography (PET) or single photon emission tomography (SPET) and intraoperative mapping by fluorescence and/or radioactive detection, using a hand-held probe or portable camera. Two main particulate pharmaceuticals are generally used for the above purposes, namely, nanocolloid of human albumin (NC) and macroaggregated albumin (MAA).

Sentinel lymph node (SLN) biopsy is a routine and well-standardized procedure in several kinds of cancer, primarily breast cancer, and melanoma. ^99m^Tc-labelled NC is the gold-standard treatment, especially in Europe. More recently, a hybrid tracer combining indocyanine green (ICG) with ^99m^Tc-labelled nanocolloid has shown additional values [1, 2], which improve SN detection not only in conventional but also in laparoscopic or robotic surgery [1, 3, 4]. The other particulate albumin agents taken into consideration are MAA. Due to their size (approximately 80 *μ*m), they embolize the first precapillary filter, previously used in pulmonary scintigraphy, and make it possible to study regional perfusion, especially in tumors. MAA labelled with a PET or SPET tracer can accurately localize the tumor and establish quantification of perfusion, while the fluorescent signal guides surgeons during tumor removal [5]. Several methods have been proposed to radiolabel near-infrared (NIR)-emitting fluorophore mannosylated-diethylene triamine pentaacetic acid (DTPA)-dextran (Tilmanocept®) with ^68^Ga and ^99m^Tc molecular imaging of SLNs [6, 7], with excellent results. However, Tilmanocept® PET scans just one patient, while our aim is to reduce costs using a commercially available, multidose kit. Recent developments in robotic surgery have highlighted the need for a specific procedure which can identify the site and contours of the target lesion [8]. The better spatial resolution of PET images helps surgeons locate the lymph node with greater precision, prior to surgery, while robotic surgery is facilitated by the fluorescence signal after injection of ICG, which enables a more accurate excision. Moreover, hybrid tracers can help to establish the dose required for the intraoperative detection of a fluorescent signature [9]. To improve these clinical applications, we have studied and prepared a trimodal targeting agent ^99m^Tc-^68^Ga-ICG-MAA or-NC based on SPET, PET, and fluorescence imaging [9]. The physical characteristics of the ^68^Ga isotope, given its high percentage of positron emission (89%), relatively short half-life (t½ = 67.71 min), and chemical properties, can label the various diagnostic molecules, making it an excellent positron emission isotope with superior resolution, speed, and quantification capacity [5]. The aim of this study was to obtain instant radiolabelled kits, integrating three imaging systems. This innovative approach would make it possible to prepare versatile, multipurpose drugs. Moreover, these radiopharmaceuticals would enable diagnostic, quantitative, preoperative PET/CT, or SPET-CT imaging and enable hybrid intraoperative mapping.

## 2. Materials and Methods

### 2.1. Materials

All the pharmaceuticals used in our studies had already been commercialized and authorized for clinical use. Nanocolloid (nanosized human colloidal particles of ≤80 nm diameter, containing 0.5 mg human albumin) is a radiopharmaceutical commercially available as NanoAlbumon® (Radiopharmacy Laboratory Ltd., Budaörs, Hungary). We tested other commercially available nanocolloid kits (Nanocoll® GE Healthcare S.r.l., Milano), but due to supply shortages in 2018, we were not able to complete the tests and report our findings. The NanoAlbumon® kit is a sterile, nonpyrogenic, lyophilized mixture of stannous chloride, glucose, poloxamer 238, disodium phosphate dehydrate E339, and sodium phytate. MAA with a diameter up to 150 *μ*m and a number of particles per vial, ranges between 2 × 10^6^ and 4.5 × 10^6^, is a commercial kit for pulmonary perfusion scintigraphy (Pulmocis® CURIUM CIS-Bio international, Saclay, France).

The kit is a sterile, nonpyrogenic, lyophilized mixture of 1.0 mg albumin aggregated with 10 mg human albumin, stannous chloride (SnCl_2_.2H_2_O), 10 mg sodium chloride, and sodium caprylate. All the reagent solutions were prepared from sterile distilled water according to European Pharmacopea. The radiolabelling procedure involved only ultrapure reagents excluding metal needles to avoid any traces of metal impurities.

### 2.2. Radiolabelling

NC or MAA commercial particle radiolabelling was performed sequentially. The kits were firstly labelled with ^99m^Tc (following the manufacturers' instructions) and then with ^68^Ga. The colabelled solutions were then tagged with the ICG fluorescent molecule. ^68^Ga and ^99m^Tc doses were assayed in a CRC-15 PET dose calibrator (Capintec, Inc., New Jersey, USA). The ^68^Ga and ^99m^Tc generators were eluted with 8 or 6 mL of HCl or saline, respectively, 24 hours prior to labelling elution so as to eliminate ^68^Zn and ^99^Tc, present in the generators and which can affect the synthesis yield.

#### 2.2.1. ^99m^Tc NC and MAA Labelling

According to the manufacturers' instructions, the NC kit was reconstituted with 1000 MBq of sodium pertechnetate [^99m^TcO_4_^−^], obtained from ^99^Mo/^99m^Tc Tekcis® generator (CURIUM IBA Cis-Bio, Gif-sur-Yvette, Paris, France) and sodium chloride per injection (0.9%). The suspension was incubated at room temperature for 25 min, according to the standard labelling procedure. Likewise, the MAA kit was reconstituted with 1000 MBq of sodium pertechnetate obtained from ^99^Mo/^99m^Tc Tekcis® generator and sodium chloride per injection (0.9%), which was then incubated at room temperature for 20 min, according to the standard labelling procedure.

#### 2.2.2. Manual ^68^Ga Labelling

^68^Ge/^68^Ga generator (1.1 GBq TiO_2_-based GalliaPharm® Eckert-Ziegler Isotope Products, Berlin, Germany) was eluted with 8 mL 0.1 N HCl (Eckert-Ziegler). 0.75 mL 0.1 N NaOH/phosphate buffer (1 mL) was added to this 0.1 N HCl solution of [^68^Ga] Ga-chloride, increasing the pH to 6–6.5. 4 mL of the resulting solution (ca. 370 MBq) was added to the ^99m^Tc-labelled kits giving a final volume of 6 mL. After mixing the solution for 30 seconds, the [^68^Ga]Ga-NC suspension was incubated in a heat block at 75°C for 15 min. The [^68^Ga]Ga-MAA suspension was incubated in a heat block at 40°C for 15 min. Only plastic needles were used as the presence of metal traces which could have seriously affected the synthesis yield.

#### 2.2.3. Automated ^68^Ga NC/MAA Labelling

To limit occupational exposure to radioactivity and ensure sterility of the radiopharmaceuticals, we also tested the results of an automated labelling system, using the ModularLab EAZY® (Eckert-Ziegler Isotope Products, Berlin) system, whose reagents can be set up under aseptic conditions. ^68^Ge/^68^Ga generator (GalliaPharm® Eckert-Ziegler 1,1 GBq) was eluted with 8 mL 0.1 M HCl. The generator was eluted 24 hours prior to synthesis to eliminate ^68^Zn, which can affect the yield. 350–700 MBq of ^68^GaCl_3_ (approximately 8.0 mL), were used in an automatic synthesis module. The ^68^Ga elution was prepurified and concentrated on an SCX column (ABX Radensberg, Germany). The purified ^68^Ga^3+^ was obtained by eluting the column with 0.8 mL of NaCl/HCl 3 M solution (pH = 1). 2 mL of CH_3_COONa/CH_3_COOH 0.8 M buffer and 400 *μ*L of EtOH/H_2_O at 50/50 % (v/v) were added to the NC lyophilized kit together with 0.8 mL of ^68^Ga^3+^ solution eluted from the column. The final volume was approximately 3.5 mL. The reactor was heated to 75 ± 2°C for 15 min. The product was diluted to 8 mL with a 0.9% saline solution. The pH of the final product ranged between 4.0 and 5.0. All chemicals were manufactured by TraceSELECT-UltraPURE from ABX Radensberg, Germany. The same procedure can be used for MAA automated labelling incubated at 40 ± 2°C for 15 min.

### 2.3. Fluorescent Tagging

Indocyanine green is a negatively charged, tricarbocyanine dye, with a molecular weight of 751.4 Da. Pharmaceutical ICG is available in a dry form (25 mg, ICG Pulsion Medical Systems, Feldkirchen, Germany), stable at room temperature and soluble in water. A vial of ICG was dissolved in 5 mL of sterile water, and its final concentration was about 6.65 mM. 100 *μ*L of this solution was added to the ^99m^Tc-^68^Ga-NC/MAA solutions. We then withdrew 500 *μ*L of the sample, which was centrifuged at 4000 rpm (12000 rpm for NC) for 10 min in a 1.5 mL vial and then washed with 500 *μ*L saline solution. The cycle was repeated 3 times.

### 2.4. NC Final Solution

The final radio and ICG-labelled solution (total volume 6 mL) contains 7.25 nmoles of NC (0.5 mg aggregated HSA), 1 GBq of ^99m^Tc (5.31 × 10^−11^ moles, 8.85 × 10^−9^ M), about 270 MBq of ^68^Ga (2.64 × 10^−12^ moles, 4.40 × 10^−10^ M), and 0.5 mg ICG (665.4 nmol, 1.11 × 10^−4^ M).

### 2.5. MAA Final Solution

The final solution (total volume 6 mL) contains 14.49 nmoles MAA (1 mg aggregated HSA), 10 mg HAS, 1 GBq of ^99m^Tc (5.31 × 10^−11^ moles, 8.85 × 10^−9^ M) about 270 MBq of ^68^Ga (2.64 × 10^−12^ moles), and 0.5 mg ICG (665.4 nmol, 1.11 × 10^−4^ M).

### 2.6. NC/MAA Final Solution Preparations Based on an Automated Synthesis Module

The final solutions (total volume 8 mL) contain 14.49 nmoles MAA or 7.25 nmoles NC, respectively: 1 GBq of ^99m^Tc (5.31 × 10^−11^ moles, 8.85 × 10^−9^ M), 270 ÷ 550 MBq of ^68^Ga (2.64 ÷ 5.28 × 10^−12^ moles), and 0.5 mg ICG (665.4 nmol, 8.32 × 10^−5^ M).

### 2.7. Quality Controls

Quality controls (QC) were performed to verify the labelling yield and stability of ^99m^Tc-^68^Ga-ICG-NC/MAA. The percentage of free ^99m^Tc and ICG was evaluated by thin-layer chromatography, using ITLC-SG (Varian, Folson–USA) as a stationary phase (15 cm long and 2 cm wide) and CH_3_OH : H_2_O 85 : 15 as a mobile phase. A 10 *μ*L spot of solution containing the sample was applied to a strip roughly 1.5 cm from the bottom edge. The strip was then placed in a separation chamber, and the solvent was run for at least 10–12 cm. Labelled NC/MAA remain at the point of application, while free ^99m^Tc pertechnetate and free ICG migrate with the solvent front. The percentage of free ^68^Ga was assessed in the same way, using 0.1 M tribasic-citrate solution, adjusted to pH 6 with HCl, as a mobile phase. NC/MAA labelled with ^68^Ga remains at the point of application, while free ^68^Ga migrates with the solvent front. According to the EU Pharmacopeia sheets, we also assessed QC of MAA labelling by filtration on polycarbonate membrane filters (3 *μ*m pore size, 25 mm diameter; Nuclepore-Whatman, GE Healthcare, Milan, Italy) and Swin-Lok® 25 mm filter holders (Nuclepore-Whatman, GE Healthcare, Milan, Italy). The fluorescence tagging yield was detected on the same ITLC-SG strip with a handheld fluorescence imager PDE probe (PhotoDynamicEye Hamamatsu by SEDA S.P.A. Italy).

### 2.8. Stability Tests

Stability assessment of the labelling and tagging compounds (MAA and NC) was performed by dilution tests in PBS (Phosphate Buffer Saline) or by challenge tests using a strong chelating agent such DTPA (diethylenetriaminepentaacetic acid).

PBS dilution test: 1 mL of compound was diluted to a total volume of 100 mL; DTPA challenge test: 1 mL of compound was diluted to 100 mL using a 1 mM DTPA solution. *In vivo* stability simulation of the trimodal labelled agents was carried out incubating 1 mL of compound in 9 mL of human serum (total volume 10 mL) for 1 and 24 hours, respectively, at 37°C. Due to ^68^Ga's short half-life, the 24 h tests cannot be performed on compounds labelled with this isotope.

### 2.9. Measurement of Nanocolloid Size

The percentage distribution (number and volume) of particle diameters was measured by dynamic light scattering (DLS) using a zetasizer particle size analyser (Malvern Instruments, Malvern, UK). This instrument has a measuring range of 0.4 nm to 8.6 *μ*m. Analysis was performed with a 90° angle over a glass cell containing 1 mL of the nanocolloidal sample at room temperature (20°C, refractive index at 578 nm = 13331, absorbance at 630 nm∼0). The nanoparticle shape was observed by electron microscopy (150000–300000x).

#### 2.9.1. Radioactivity Distribution after ^99m^Tc Labelling

Nanofiltration under vacuum is the method used to determine radioactive distribution in various sizes of nanocolloidal particles. Polycarbonate membrane filters (50 and 30 nm pore size, 25 mm diameter; Nuclepore-Whatman) and Swin-Lok 25 mm filter holders (Nuclepore-Whatman) were used. A 0.1 mL sample of ^99m^Tc-^68^Ga-ICG-NC was passed through the cascade of two filters and rinsed with 5 mL of N_2_ purged milliQ water (Milli-Q System, Merck Millipore, Darmstadt, Germany). Filtered radioactive fractions (as percentages) were determined using a 3 × 3  inch NaI(Tl) pinhole 16 × 40 mm gamma counter (Raytest, Straubenhardt, Germany). An overpressure of N_2_ at the top of the filters made it possible to exclude the presence of oxygen and enable a faster filtration procedure [10].

### 2.10. Measurement of MAA Particle Size

All tests were performed using the Pulmocis® commercial kit, as it is the only MAA product available in Italy as of October 2018. Optical microscopy measured the particle size and number per vial, using a Burker-Turk cell counting chamber. The measurements were performed after ^99m^Tc and ^68^Ga labelling, at 40°C. ImageJ® (National Institute of Health—USA) software was used for image processing and measurements.

### 2.11. *Ex Vivo* Experiment

To simulate breast surgical procedures, an *ex vivo* test was performed after total mastectomy. The tests were performed in two operating rooms, one equipped with halogen operating lamps and the other with LED lighting lamps. 300 *μ*l (50 MBq) of ^99m^Tc-^68^Ga-ICG-MAA were injected into a selected site of the breast. At the same time, 300 *μ*l of free ICG (prepared according to SPC's pharmaceutical indications) were injected into another area of the breast.

## 3. Results

### 3.1. Radioisotope Purity

Radioisotope purity measurement was assessed after ^68^Ga decay, with a 3″ × 3″ NaI(Tl) pinhole gamma counter. The ^68^Ge percentage found in the final product was <0.001 % of the total radioactivity, according to the Ph.Eur. for ^68^Ga-radiopharmaceuticals.

### 3.2. Labelling Yield and Quality Control: Radiochemical and Fluorescent Purity

**Figure 1:**
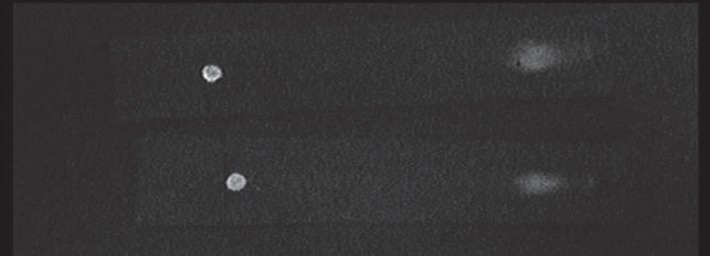
ITLC strips (picture of PDE output). ICG fluorescence remains mostly at the MAA-labelled point of application even though about 10 % of the dye was found free in the solution.

**Figure 2:**
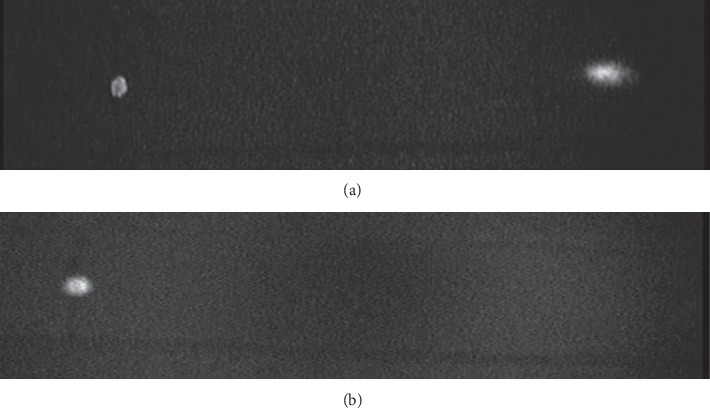
ITLC strips (pictures of PDE output). Fluorescence of NC-labelled IGC before (a) and after (b) washing with saline solution. Physiological saline solution washing simulates lymphatic drainage *in vivo*. In figure (b), the free dye is completely removed.

**Figure 3:**
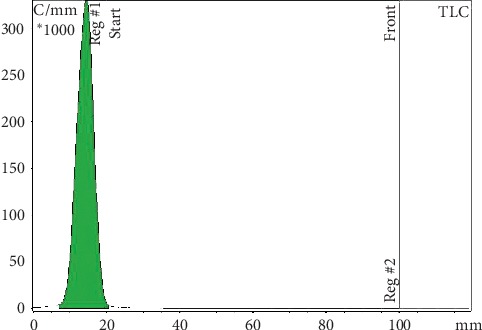
ITLC radiochromatogram: ^68^Ga-^99m^Tc-labelled MAA remains at the point of application. Free ^99m^Tc pertechnetate migrates with the solvent front. Mobile phases for ^99m^Tc analysis: MetOH : H_2_O 85 : 15.

**Figure 4:**
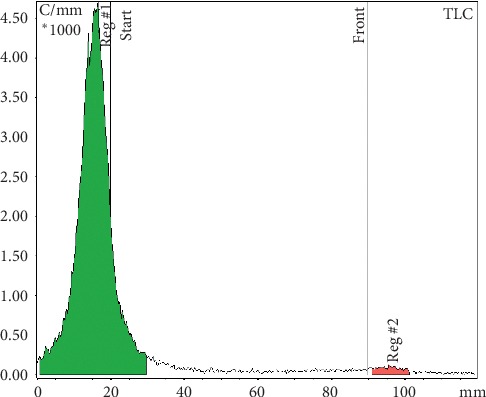
ITLC radiochromatogram: MAA labelled with ^68^Ga remains at the point of application. Free ^68^Ga at the solvent front. Mobile phases for ^68^Ga analysis: tribasic-citrate solution 0.1 M, pH 6.

**Figure 5:**
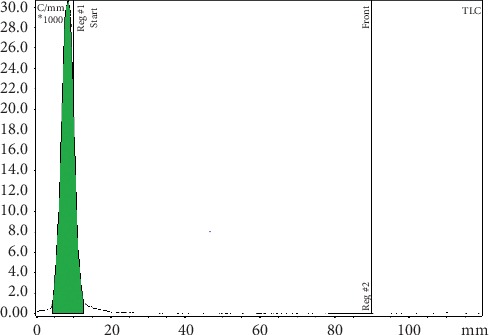
ITLC radiochromatogram: ^68^Ga-^99m^Tc-labelled NC remains at the point of application. Free ^99m^Tc pertechnetate migrates to the solvent front. Mobile phases for ^99m^Tc analysis: MetOH : H_2_O 85 : 15.

**Figure 6:**
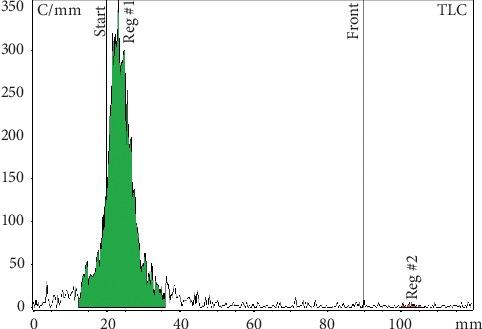
ITLC radiochromatogram: NC labelled with ^68^Ga remains at the point of application. Free ^68^Ga migrates with the solvent front. Mobile phases for ^68^Ga analysis: tribasic-citrate solution 0.1 M, pH 6.

The radiolabelling yield and radiochemical purity for the ^99m^Tc-^68^Ga-labelled MAA and NC (Table 1) were always higher than 97%, as measured by instant TLC. The ITLC strips did not reveal radioactivity at the solvent front (RF∼8 cm). The percentages of each fraction were determined relatively to the total activity of the chromatogram. Similarly, the ICG-tagged MAA and NC remained at the point of application on the ITLC strips and the free ICG migrates at the solvent front (Figures 1–6). No significant differences were detected during the QC of MAA for free ^99m^Tc/^68^Ga, when it was filtered with 3 *μ*m polycarbonate filters. The reaction yield is significantly affected by pH and must be controlled. pH values <3 and >10 give very poor labelling results.

### 3.3. Radiochemical and Fluorescent Stability Tests

Dilution tests in PBS and challenge tests with DTPA of both MAA and NC compounds labelled with ^68^Ga, ^99m^Tc and ICG show a radioactive and fluorescence distribution comparable with undiluted controls (Tables 2 and 3). A negligible radioactivity/fluorescence trace (<5%) was detected at the solvent front on ITCL-SG strips developed with tribasic-citrate 0.1 M. As a result, the percentage of the bound products is always over 95%. ^99m^Tc-^68^Ga-IGC-MAA exhibited good stability both after 1 h and 24 h incubation with human serum for all labelling agents. ^99m^Tc-^68^Ga-IGC-NC also exhibited good stability after 1 hr and 24 hr incubation for ^99m^Tc labelling and IGC tagging agents, while the stability of the bond with ^68^Ga dropped to 80% after 1 hr incubation.

### 3.4. Measurement of Particle Size in NanoAlbumon® Commercial Kits

**Figure 7:**
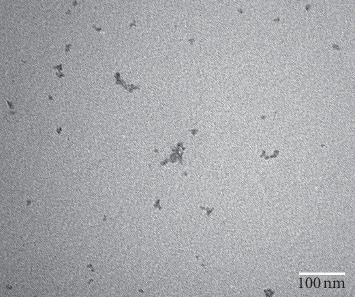
NanoAlbumon® commercial kit (TEM 300000 x).

Particle diameter ranges between 12 and 122 nm. 30% of the total number of particles were found to have a diameter of about 18 nm (SD = 3 nm), and 99% have a diameter of <33 nm [10]. The nanoparticle shape, observed by electron mycroscopy, is depicted in Figure 7.

### 3.5. Radioactivity Distribution vs. Nanocolloid Size after ^99m^Tc/^68^Ga Labelling and ICG Tagging

Radioactivity distribution data revealed that for ^99m^Tc-NC-HSA commercial kits, less than 10% of radioactivity bound to particles with a diameter <15 nm [10]. Maximum radioactivity (∼50%) was bound to particles with a diameter between 30 and 50 nm. Tables 4 and 5 report the percentage of labelling values for particles with a diameter of 30 and 50 nm. The labelling and tagging procedures did not substantially influence radioactivity distribution between the three dimensional fractions.

### 3.6. Particle Size Measurement of an MAA Commercial Kit

**Figure 8:**
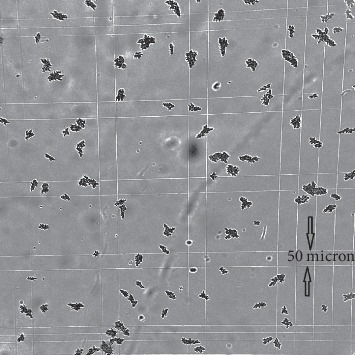
Pulmocis® commercial kit incubated at 40°C for 15 minutes (optical microscopy 100x).

**Figure 9:**
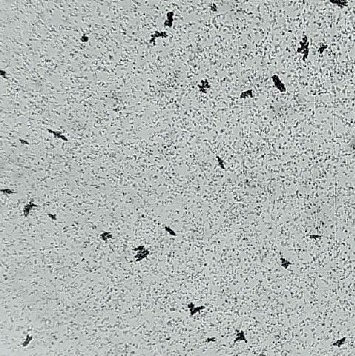
Pulmocis® commercial kit incubated at 75°C for 15 minutes. The macroaggregate disaggregation is evident, forming subunits of about 7 *μ*m (optical microscopy 100 x).

Optical microscopy revealed that the Pulmocis® MAA commercial kit consisted of a mixture of irregular particle aggregates whose dimensions ranged between 7 and 100 *μ*m, with a prevalence of particles of about 50 *μ*m (Figure 8). Macroaggregate counts averaged about 2765000 MAA particle/vial, in conformity with the manufacturer's specifications (2–4 × 10^6^ particles/vial). We started measuring the particle size of MAA commercial kits labelled with^68^Ga after 15 min incubation at 75°C, as reported in the literature [11–14]. However, optical microscopy revealed a substantial modification in MAA particle size and structure. In fact, when heated to 75°C, a fraction of the macroaggregates disaggregated into subunits of about 7 ± 2 *μ*m (Figure 9). After being centrifuged at 300 rpm × 5 min, this fraction contained an amount of total radioactivity of about 5.5%. Therefore, we decided to reduce the^68^Ga labelling temperature from 75°C to 40°C. This preserved the particles' structure and size, as well as labelling yield (>99%) and stability parameters, that were virtually unchanged (Table 3 and Figure 8).

### 3.7*. Ex Vivo* Specimen Analysis

**Figure 10:**
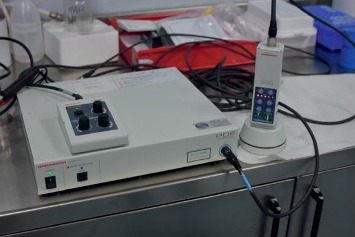
PDE probe (PhotoDynamicEye Hamamatsu by SEDA S.P.A. Italy) handheld fluorescence imager.

**Figure 11:**
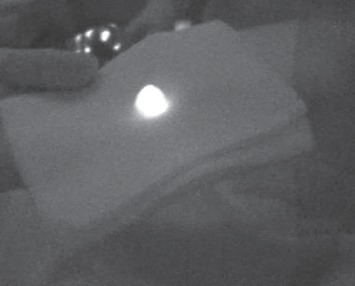
NC-IGC tagged excised lymph node (*ex vivo*).

**Figure 12:**
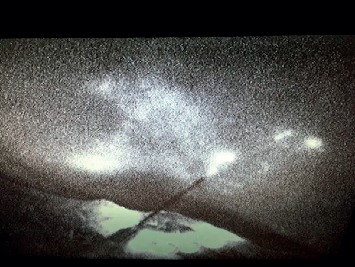
Site of ICG solution injection. After intraparenchymal administration in a breast tissue specimen, the ICG solution rapidly spreads into the surrounding tissue (*ex vivo).*

**Figure 13:**
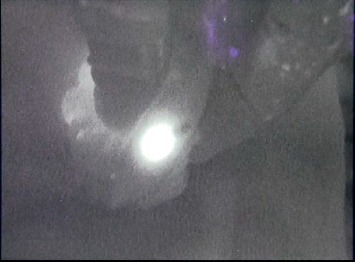
Site of MAA ICG-tagged injection. After intraparenchymal administration in breast tissue, ICG-tagged MAA remains confined in the injection site without spreading (*ex vivo*).

Our goal was to compare tissue perfusion of free ICG versus ICG bound to ^99m^Tc and ^68^Ga-labelled MAA. The ex vivo images were obtained with a PDE handheld fluorescent imaging probe (Figure 10). Fluorescence imaging was used to visualize the localization and distribution of the tracers after injection into breast and lymphoid tissues (Figure 11). The ex vivo experiments showed that free ICG diffusion into the surrounding area is rapid, aspecific, and extends over several centimeters (Figure 12), making its use impossible in identifying the tumor. On the contrary, the ICG-tagged MAA preparation remains closely confined to the injection area, which can therefore be accurately identified and excised (Figure 13). Furthermore, the ex vivo experiments showed that this tracer may also be useful in anatomopathological diagnoses. In fact, the radioactive signal rapidly decays, while the fluorescent one can be detected for several days, such as in the lymph nodes as well as in the removed tissue samples.

## 4. Discussion

Multimodality imaging is a relatively new concept that is changing the field of clinical imaging. The combination of radionuclides with fluorophores makes it possible to assess and localize the disease using SPET/PET-CT or MRI. Thereafter, intraoperative localization is facilitated with imaging or nonimaging probes which can detect the radioactive and/or optical signal. This approach requires agents that are able to emit both nuclear and optical signals [15–17].

Several examples of a bimodal approach are present in the literature [1, 2, 18–20], while to our knowledge, this is the first attempt to label HSA particles using a tri-modal method.

Our results also confirm that it is possible to obtain a trimodal tracer using a single commercial kit developed for ^99m^Tc labelling (albumin macroaggregates and nanocolloidal human albumin) and available ICG and ^68^Ga chloride sterile precursors. The kit was firstly dual-radiolabelled with ^68^Ga and ^99m^Tc and then tagged with fluorescent ICG [9].

This approach poses two problems. The first concerns the technical compatibility of the labelling methods using the same kit without altering its chemical composition, while the second involves practical considerations regarding the use of this procedure in routine radiopharmacy and clinical practice.

Several attempts have been made to label MAA commercially available kits with ^68^Ga, which excluded technetium labelling [21], avoiding the use of an intermediate chelator. Radiometals are typically bound to nanoparticles via chelation [22]. However, this approach was limited to pilot studies regarding albumin particles [12, 13]. More recent research, including our own findings, indicates that an efficient ^68^Ga labelling does not require preconjugation with DOTA or other chelators.

The chemical nature of ^68^Ga binding to NC/MAA is not fully known. One hypothesis involves the absorption of insoluble hydrolyzed gallium hydroxide on the surface of NC/MAA. On the contrary, a trapping mechanism due to specific interactions of the Ga^3+^ ions with protein lone pairs exposed at the particle surface cannot be excluded [11]. pH is an essential parameter influencing Ga binding to NC/MAA with an optimal range between 3.5 and 6.5 [3, 11–13] as confirmed by our results. In all likelihood, the protein structure does not favour the binding of the GaCl_4_^−^ complex in strong acidic conditions. Neutralisation of the ^68^Ge/^68^Ga generator eluate might gradually increase the affinity of the Ga(OH)_2_^+^, Ga(OH)^2+^, and Ga(OH)_3_ chemical species for NC/MAA.

Incubation temperature was a more important factor. Initially, we followed the indications of Mueller et al. [14], who obtained a ^68^Ga-MAA labelling yield of about 99 ± 0.5%, after 15 minutes of incubation at 75°C, at pH 6. However, as highlighted in the results, the 75°C incubation of Pulmocis® leads to the partial loss of MAA structure and to a drastic dimensional decrease from 50 *μ*m to less than 7 *μ*m. Since the dimensions of these MAA subunits are comparable with capillary diameters, we hypothesized that the temperature-dependent fragmentation of the particles could decrease MAA's embolic efficacy, compromising several *in vivo* diagnostic applications of these compounds. In fact, these small fragments can carry a significant amount of radioactivity. Fragments smaller than 10 *μ*m escape the capillary filter and are taken up by the RES in spleen, liver, and bone marrow, thus deteriorating the target to background ratio and hampering a quantitative approach. Shanehsazzadeh et al. [3] reported that, after centrifugation, about 1.31% of MAA particles had a diameter of less than 2 *μ*m and 5.2% a diameter between 2 and 10 *μ*m, resulting in an unwanted uptake in liver and kidneys and an approximately 0.6% undesirable bone marrow uptake, when tested in mice.

An incubation temperature of 40°C avoids the abovementioned phenomenon as the labelling yield remains higher than 99%, with no remarkable differences in the stability tests.

Comparing our data with the literature, we hypothesise that size and morphology differences are related to the MAA commercial kit used and closely linked to labelling temperature. Maus et al. [8] did not observe any fragmentation using the MAA GE kit (Maasol) at 95°C, but as a coauthor of another study led by Shanehsazzadeh et al. [3], he too reported fragmentation at 75°C, when using the Pars-Isotope Company kit (TCKPars-1800). Nonetheless, particle size and morphology after ^68^Ga labelling has not been exhaustively elucidated in the literature. Only six groups focused on this issue [3, 8, 11, 14–16]. Four of them [8, 11, 14, 16] did not observe any modification of particle size and morphology either with optical microscopy or SEM [8], while the other two groups [3, 15] confirm MAA fragmentation after labelling, as observed in our experience.

Less information is available in the literature regarding ^68^Ga labelling of HSA nanocolloid. Maus's attempts [8] to label nanocolloid (Nanocoll®) gave poor results due to a likely agglutination of particles which changed their size. After intravenous injection in rats, resulting particles were prevalently sequestered in the lungs rather than in the liver. The authors attributed this agglutination to the reaction temperature of 75°C. The agglutination observed by Maus et al. [8] might be due to the prelabelling washing step and removing the Poloxamer 358 present in this formulation. On the contrary, we did not observe any significant structure or size modifications of the NC particle after ^68^Ga labelling for 15 min at 75°C. In fact, Nuclepore filtration of NanoAlbumon® showed only a negligible size variation after ^68^Ga labelling and confirmed the data of our previous report [10].

ICG was the third step of the labelling procedure. ICG must first be dissolved in water (5 mg/mL) and then diluted in physiological saline, as it is not readily soluble in this solution. Tagging yield and stability tests confirm its robust and stable bonding with MAA and NC. The amount of free fluorophore, observed by ITLC after labelling, does not condition its *in vivo* applications, due to its short biological half-life in the human body, as demonstrated by other authors [18]. Several clinical trials have used ^99m^Tc-ICG-NC without evaluating the ICG percentage bound to NC [19]. Free ICG might bind to lipid lipoprotein complexes (*β*-lipoprotein) [20], resulting in a more intense fluorescence than ICG-bound to free cholesterol [21]. Its binding to blood proteins gradually shifts IR absorption and emission peaks toward longer wavelengths [22].

Previous studies [3, 8, 11, 13] have highlighted the importance of the prewashing step prior to ^68^Ga labelling, so as to remove Sn(II), free albumin, surfactants and other coformulants present in the commercial kit. We intentionally omitted this prewashing step firstly because the kit needs to be labelled with ^99m^Tc and secondly because Mueller et al. [14] reported no significant differences in ^68^Ga's radiolabelling yield using the excipient-free and original MAA kit.

We did not perform postpurification of our compounds by centrifugation to discard any free ^68^Ga and ICG [3, 8] because ^68^Ga's labelling yield was always ≥97%, and the amount of free ICG does not limit its *in vivo* use due to its short biological half-life [23]. Not only did this procedure allow us to reduce labelling time but also the risk of microbial contamination during the washing procedure and reduced occupational exposure.

The stability of ^99m^Tc-^68^Ga-ICG-NC/MAA compounds was confirmed by testing their binding in diluted solution, in challenge test with DTPA and incubation in human serum. The coordination reaction equilibrium between the metal ions and the NC/MAA particles shifts toward dissociation upon dilution, releasing radioactive metals. In our studies, the labelling yield showed no significant signs of deterioration in PBS test dilution. As the acyclic ligand DTPA complexes gallium with high affinity, we used it to strip the less stable, nonspecific bound Ga-ions from the compound. The resulting loss of radioactivity is negligible, albeit slightly more evident in NC than in MAA, but does not potentially affect its *in vivo* behaviour. Human serum stability, at 37°C for at least one hour, simulates physiological conditions after subcutaneous or intraparenchymal administration. All the labelled compounds tested confirm their stability in serum, except for NC which reveals a significant loss of ^68^Ga. This loss might not prove to be a limit in a clinical setting, because unbound Gallium (Ga^3+^) is rapidly removed by circulating lymph.

*Ex vivo* stability in mammary tissue further confirmed their *in vitro* stability, even though this *ex vivo* model does not take into account other additional parameters such as circulating blood.

Another issue regarding the use of multimodal tracers involves stability interference due to different labelling which might alter their biodistribution. In theory, irradiation can induce radiobleaching of the dye. However, this does not occur when using multimodality tracers immediately after their preparation, and therefore as in our formulation, they do not influence *in vivo* biodistribution [24].

Our proposed kit labelling procedure can be adjusted to meet specific clinical needs, using one, two, or three imaging components. van Leeuwen et al. [20] summarized and compared the different tracers used for SLN procedures, highlighting the benefits of the hybrid tracer.

ICG cannot be discerned by the human eye. Lighting used in operating theatres can interfere with IR probe-acquired imaging (PDE). If the lighting has a strong emission in the infrared spectrum, when using conventional halogen lamps, the ICG signal cannot be distinguished from the background. LED surgical lamps with an almost monochromatic light emission in the yellow-green spectrum avoid this problem.

Another problem related to free ICG alone is the attenuation in the overlying tissue. In fact, a target located deeper than 0.5–1 cm cannot be easily detected. Other authors [25, 26] deem lesion detection to be inadequate when deeper than 2 cm, while the radioactive signal is still clearly detectable. In addition, the nonquantitative nature of fluorescence emission is an further impediment when using ICG as a single tracer. Furthermore, in other body regions such as head and neck or pelvic area, where lymphatic drainage shows wide interpatient variability, additional problems are involved [27, 28].

When a SLN is located in close proximity to the injection site, its activity may be masked by the larger amount of the administered radioactivity. This so-called shine-through phenomenon limits preoperative detection with planar scintigraphy or SPET-CT [29]. The use of PET-CT, with better spatial resolution, might avoid this problem. In addition, the opportunity to quantify the absolute target uptake in terms of SUV (standardized uptake value) allows clinicians to predict intraoperative detectability. During operative exploration, the fluorescent signal is better located and clearly discriminates the target from the injection site, as shown by the *ex vivo* experiment.

Table 6 summarizes the pros and con factors of the different labelling techniques in pre- and intraoperative settings.

In comparison to *γ* and *β*^−^ emitters, *β*^+^ isotopes are less frequently used in radioguided surgery due to the large amount of 511 keV annihilation *γ* rays, giving rise to several problems. Firstly, it is difficult to collimate these photons, and secondly because it increases operators' radiation exposure. An alternative approach may be the use of dedicated positron detectors which are substantially transparent to 511 keV photons, thus avoiding the limits of collimation and depth assessment of the target. Recently Collamati et al. [30] experimented a p-terphenyl-based probe prototype, which can be successfully used with ^68^Ga and other *β*^+^ isotopes. ^68^Ga's short half-life requires a thorough planning process from tracer administration to PET scan and patient transport to the operating room [31, 32].

Another practical application of our trimodal compounds might be their use in robotic surgery. Identification of the exact site of the lesion is greatly enhanced if ICG fluorescence is combined with a radioactive signal from the same probe [33, 34]. In particular, the Da Vinci robot can be equipped with a fluorescence imager and radioactivity detection system, thus avoiding problems related to the use of halogen lamps, as occurs during open surgery. Very few recent studies have investigated the potential role of PET/CT-guided robot-assisted surgery and robotic-arm PET/CT-assisted biopsy [5, 32]. Although further studies and validation testing must be carried out before their clinical use, we are confident that the ^99m^Tc-^68^Ga-ICG-NC/MAA tracer can also be used to great advantage in robotic surgery.

## 5. Conclusions

This first attempt shows the feasibility of obtaining a trimodal imaging (*γ*/*β*^+^/fluorescence) agent using a commercially available kit of albumin macroaggregates or nanocolloids, using a simple procedure which does not require additional steps such as centrifugation and pre- or postpurification.

## Figures and Tables

**Table 1:** Nanocolloids and macroaggregates: labelling and tagging yields.

	Nanocolloids	Macroaggregates
^99m^Tc bound %	>99.0	>99.0
^68^Ga bound %	97.0 ± 1	97.0 ± 1
ICG bound %	≥98	≥98

**Table 2:** Nanocolloid stability tests.

	DTPA 1 h	Serum 1 h	Serum 24 h	PBS dilution
^99m^Tc bound %	97.6 ± 1	97.2 ± 1	89.0 ± 5	96.7 ± 1
^68^Ga bound %	95.0 ± 2	80 ± 10	—	—
ICG bound %	≥98	≥98	≥97	≥97

**Table 3:** Macroaggregate stability tests.

	DTPA 1 h	Serum 1 h	Serum 24 h	PBS dilution
^99m^Tc bound %	99.0 ± 1	99.0 ± 1	99.0 ± 1	98.0 ± 1
^68^Ga bound %	96.0 ± 2	94 ± 3	—	—
ICG bound %	≥98	≥97	≥97	≥97

**Table 4:** Radioactivity distribution data after^99m^Tc labelling.

Radioactivity (%)	
Ø particle (nm)	NanoAlbumon®
Ø > 50	25
30 < Ø < 50	55
0 < Ø < 30	20

Ø, diameter of particles; CI (95%) = ±5%.

**Table 5:** Radioactivity distribution data after^99m^Tc, ^68^Ga labelling, and ICG tagging.

Radioactivity (%)	
Ø particle (nm)	NanoAlbumon®
Ø > 50	24
30 < Ø < 50	62
0 < Ø < 30	14

Ø, diameter of particles; CI (95%) = ±5%.

**Table 6:** Pros and cons of nanocolloid with different labelling method in the pre- and intraoperative setting.

Nanocolloid label and detection	Preoperative imaging		Operative detection	
	Pros	Cons	Pros	Cons
^99m^Tc planar	(i) Well-assessed methodology	(i) Shine through phenomenon (ii) Gross anatomic landmark	(i) 1 or 2 days protocol possible (ii) 6-hour half-life enables *ex vivo* quantification of excised SLN (iii) No tracer spread during surgery (iv) Portable cameras available	(i) Difficulty to assess the depth of the signal
^99m^Tc SPET-CT	(i) Good anatomical localization (ii) Shine through phenomenon less relevant	(i) Absolute quantification less accurate than with PET		

^68^Ga PET-CT	(i) Precise anatomical localization of the target (ii) Better differentiation between the first and second echelon lymph nodes (iii) Accurate quantification of the uptake (SUV)	(ii) Logistics	(i) Electronic collimation needed for gamma detection (ii) New positron detectors assuring high sensitivity for superficial targets	(i) Thorough logistic organization (ii) Radioprotection problems (iii) Difficult depth assessment with gamma ray detectors

ICG		(i) Poor quality detection strictly depth-dependent	(i) High contrast and sensitivity, low noise background (ii) Long-term signal availability up to many days (iii) Easy use	(i) Obesity (ii) Autofluorescence (iii) The amount of fluorophore within tissue cannot be determined accurately by fluorescence intensity measurements

## Data Availability

The laboratory experimental data used to support the findings of this study are available from the corresponding author upon request.
